# Species‐specific effects of production practices on genetic diversity in plant reintroduction programs

**DOI:** 10.1111/eva.13614

**Published:** 2023-11-20

**Authors:** Zoe Diaz‐Martin, Marcello De Vitis, Kayri Havens, Andrea T. Kramer, Linda M. MacKechnie, Jeremie Fant

**Affiliations:** ^1^ Department of Biology Spelman College Atlanta Georgia USA; ^2^ Chicago Botanic Garden Negaunee Institute for Plant Conservation Science and Action Glencoe Illinois USA; ^3^ Plant Biology and Conservation Northwestern University Evanston Illinois USA; ^4^ Southeastern Grasslands Institute Austin Peay State University Clarksville Tennessee USA; ^5^ Ball Horticultural Company West Chicago Illinois USA

**Keywords:** genomics, plant conservation, plant propagation, seed dormancy, seed germination, wild violets

## Abstract

Plant production practices can influence the genetic diversity of cultivated plant materials and, ultimately, their potential to adapt to a reintroduction site. A common step in the plant production process is the application of seed pretreatment to alleviate physiological seed dormancy and successfully germinate seeds. In production settings, the seeds that germinate more rapidly may be favored in order to fill plant quotas. In this study, we investigated how the application of cold‐moist stratification treatments with different durations can lead to differences in the genetic diversity of the propagated plant materials. Specifically, we exposed seeds of three *Viola* species to two different cold stratification durations, and then we analyzed the genetic diversity of the resulting subpopulations through double‐digestion restriction site‐associated sequencing (ddRADseq). Our results show that, in two out of three species, utilizing a short stratification period will decrease the genetic diversity of neutral and expressed loci, likely due to the imposition of a genetic bottleneck and artificial selection. We conclude that, in some species, the use of minimal stratification practices in production may jeopardize the adaptive potential and long‐term persistence of reintroduced populations and suggest that practitioners carefully consider the evolutionary implications of their production protocols. We highlight the need to consider the germination ecology of target species when selecting the length of dormancy‐breaking pretreatments.

## 1 INTRODUCTION

Humans have influenced the genetic composition of other species for millennia, as exemplified by the domestication of crops and animals (Diamond, 2002; Driscoll et al., 2009; Meyer & Purugganan, 2013). Typically, domestication is focused on selection for heritable traits that favor productivity, and it is often coupled with decreased genetic diversity (Harlan, 1992; Pizza et al., 2021; Smýkal et al., 2018) as well as a dependence on human oversight (Pingali, 2012). However, for species where the goal is reintroduction to the wild, the modification of traits and decrease in genetic diversity associated with cultivation practices could be detrimental to species long‐term persistence (Frankham, 2008; Marsden et al., 2016). The negative impacts of such changes have been well documented in wild animals that are captive raised for re‐release back into the wild, such as fish stocks reintroduced into streams and rivers (Araki et al., 2007; Christie et al., 2012). Similarly, for nonagricultural plant species, the production of plant material for restoration is expected to occur at large scales, in a short amount of time, and at the lowest possible cost (Leger et al., 2021). As a result, the pressure to meet production goals can impose unintended selection, genetic bottlenecks, and selective sweeps (Semagn et al., 2021) that can favor homogeneity in traits (Ensslin et al., 2018; Lauterbach et al., 2012), cause shifts in allele frequencies (Dyer et al., 2016; Kucera et al., 2022), and lead to decreases in fitness in natural conditions (Pizza et al., 2021). Ultimately, production can lead to changes in genetically controlled traits and the reduction of genetic diversity (Basey et al., 2015; Conrady et al., 2022, 2023; Espeland et al., 2017; Leger et al., 2021; Nagel et al., 2019; Pizza et al., 2021), which is likely to reduce the success and effectiveness of reintroduction efforts (Frankham et al., 2010). Indeed, for many plant species, genetic diversity is significantly correlated with higher fitness (Leimu et al., 2006). Although the theory is well‐established, there are few studies illustrating how the application of specific production practices can influence the genetic diversity of cultivated plant materials and their suitability for reintroduction efforts (see Conrady et al., 2022, 2023).

**FIGURE 1:**
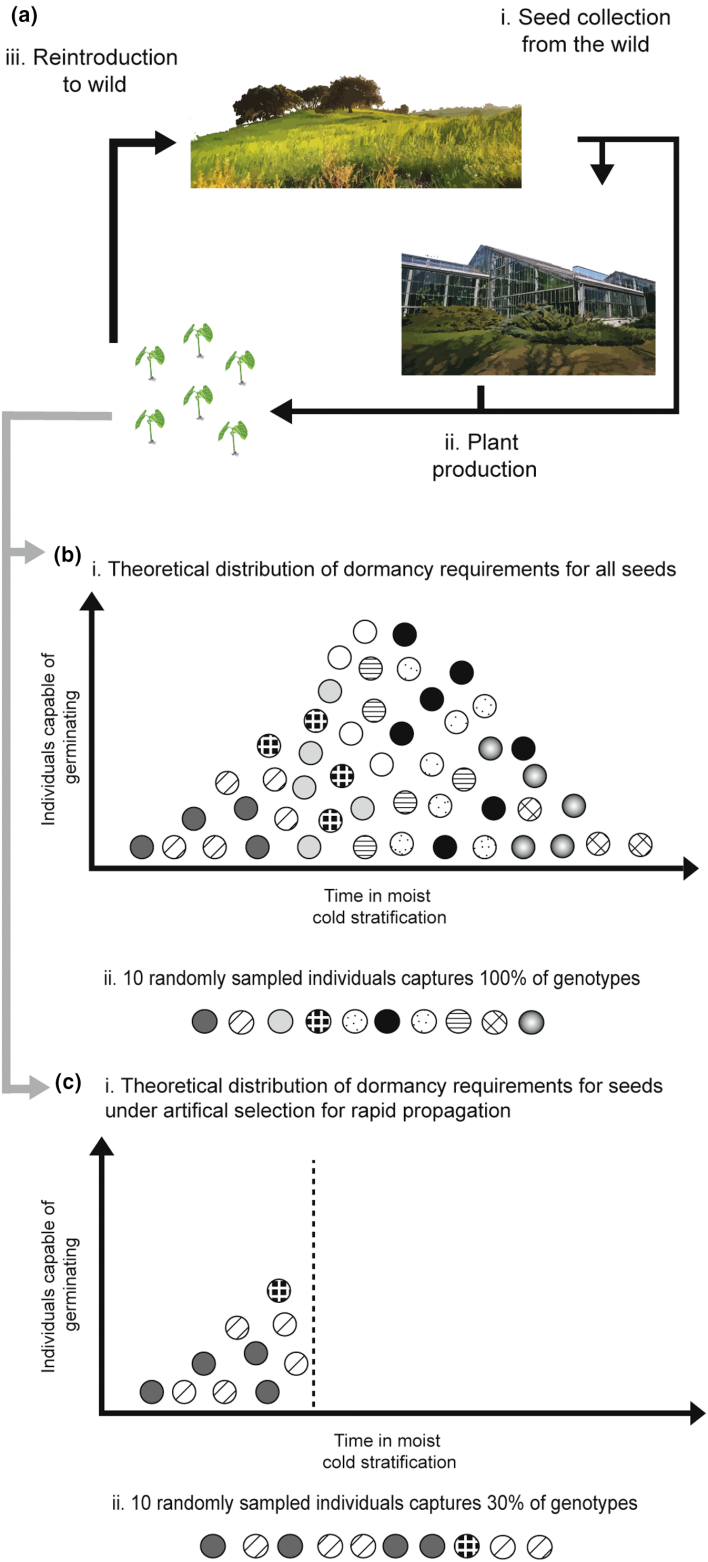
Conceptual diagram of how horticultural production practices may decrease genetic diversity. Seeds are collected from wild populations and germinated in nurseries or commercial companies to propagate plants for reintroduction (ai‐iii). In theory the distribution of dormancy requirements will differ for individuals with differing genotypes (filled circles) with some germinating under shorter cold stratification and others requiring longer cold stratification (bi). A random sample of ten individuals would largely represent the allele frequencies occurring in the population—genotypes regulating dormancy breakage are also linked to variety of genotypes regulating other biological functions (bii). In horticultural production settings, artificial selection for rapid germination would favor individuals that require a shorter cold stratification period (ci), which may represent only a subset of allele frequencies of the total population (i.e., a genetic bottleneck) and select for genotypes related to rapid germination (i.e., artificial selection) which may be linked to loci representing a subset of genotypes (i.e., a selective sweep) (cii). Propagated individuals are then reintroduced into wild populations (aiv).

A common practice in plant reintroductions is to propagate plugs (i.e., plants) starting from seeds sourced either directly from wild plant populations or from seed production facilities (Pedrini et al., 2020) (Figure 1ai–aii). In both cases, a critical step in the propagation process is breaking seed dormancy to allow germination, which in temperate species often requires a cold‐moist stratification pretreatment to alleviate physiological dormancy (Baskin & Baskin, 2014). The length of the cold stratification needed to break dormancy can vary by species but can also differ between populations of the same species as well as between individual seeds within a population (Baskin & Baskin, 2014; Bradford & Bello, 2022; Kildisheva et al., 2020) (Figure 1bi). In production settings, the seeds that germinate quickly are typically favored in order to fill plant quotas (Basey et al., 2015). For example, if a cold stratification of 30 days results in a satisfactory 50% germination rate, practitioners might only apply this duration, even if a longer cold stratification of 90 days would result in 80% germination. The use of propagation practices that apply a short cold stratification could affect genetic diversity and influence the capacity of produced plant materials to break seed dormancy in a variable environment.

The interaction between genotypes and the environment plays an important role in the complex process of breaking seed dormancy (Finch‐Savage & Leubner‐Metzger, 2006; Yan & Chen, 2020). Seed dormancy is a heritable and complex trait (Kishchenko et al., 2020; Long et al., 2015), for which the genetic background of individuals interacts with the environment to influence dormancy requirements. Cues in both the maternal and offspring environment, such as temperature and light, regulate the alleviation of seed dormancy (Chiang et al., 2011; Footitt et al., 2013; He et al., 2014; Kerdaffrec & Nordborg, 2017). Additionally, there are a variety of gene pathways relating to the hormonal balance of gibberellic acid (GA) and abscisic acid (ABA) that interact to impact dormancy breakage (Finch‐Savage & Leubner‐Metzger, 2006; Kishchenko et al., 2020; Tuan et al., 2018). In natural plant populations, variability in seed dormancy and germination can be an advantageous bet‐hedging strategy that ensures germination across a range of abiotic and biotic conditions to ensure the population persistence in heterogenous environments (Long et al., 2015). As seed dormancy has a genetic underpinning that interacts with the environment, retaining genetic diversity in wild populations is an important part of maintaining a species' ability to adapt to changing selective pressures.

Given the relationship between plant genotypes, the environment, and seed dormancy, the production practices used to propagate plants for restoration could have unintended consequences on the genetic makeup of the generated plant materials. One mechanism explaining a change in genetic diversity in produced plants could be a genetic bottleneck where only a subset of genotypes from the source population is included and, therefore, represented in the plant material available for reintroduction (Kettle et al., 2008) (Figure 1). In addition, when there is genetic variation among individuals linked to differences in breaking seed dormancy (Figure 1bi), production practices could also lead to favoring genotypes that require a short cold stratification to break their dormancy (Figure 1c,b). This imposition of artificial selection may not only change the genetic composition of the population for this trait but could also result in a selective sweep whereby loci linked to gene regions associated with rapid germination are also selected (Meyer & Purugganan, 2013; Pizza et al., 2021). Taken together, if the dormancy‐breaking trait is controlled, at least in part, by genetic factors, then selective sweeps and genetic bottlenecks are likely to reduce genetic diversity in the cultivated populations ultimately destined for in situ conservation activities (Stephan, 2019; Willi et al., 2022). Such a decrease in genetic diversity could negatively impact the establishment or persistence of reintroduced populations since genetic diversity is linked to the population's evolutionary potential, or ability to respond to variable environments (Attard et al., 2016; Ren et al., 2014). On the other hand, if the dormancy‐breaking trait is mainly controlled by a plastic response and genotype × environment interactions, then genetic diversity of produced material should be the same regardless of whether practices use a short or long cold‐stratification approach (Ensslin et al., 2018; Kucera et al., 2022). Evaluating if production practices impact levels of genetic diversity in plant materials destined for reintroduction efforts will help refine best practices for conservation efforts.

To investigate how the application of a short cold stratification might impact the genetic composition of the plants produced for reintroduction efforts, we took a genomics approach to compare the gene diversity (H_S_) and allelic richness (A_R_) of three species of violets (*Viola pedatifida*, *V. sagittata*, and *V. lanceolata*) that germinated after being exposed to cold stratification of different lengths. Violets are often the target of reintroduction efforts in Midwestern United States prairie habitats and exhibit seed dormancy traits that have posed a challenge to achieving optimal germination for propagation purposes (Kilgore et al., 2022). We germinated seeds sourced from local conservation nurseries by exposing them to either a short or long cold stratification treatment for a single generation in controlled conditions. We assessed the genetic diversity of each group, or subpopulation, using ddRADseq (Peterson et al., 2012) and hypothesized that the subpopulations germinated through the short stratification treatments would display lower genetic diversity than the long stratification pretreatment subpopulations. Our work advances our understanding of how the application of specific production practices may have unintended consequences for the species involved in restoration efforts.

## 2 MATERIALS AND METHODS

### 2.1 Study species

Species in the genus *Viola* are of high restoration interest in the Midwest U.S. because they provide important early‐spring resources for pollinators and some violet species are the exclusive larval hosts for the endangered regal fritillary butterfly (*Speyeria idalia*) (Selby, 2007). To address the drastic decline of the regal fritillary butterfly, efforts have been undertaken to reintroduce violet populations into prairie restorations, but establishment success is often low (personal communication, Alyssa Nyberg, The Nature Conservancy). Violet conservation activities are still limited by a lack of information on optimal seed germination protocols (Kilgore et al., 2022). Beyond the presence of a physiological dormancy in temperate violets, the optimal duration of the cold stratification required for maximum germination is still unknown for many *Viola* species (Kilgore et al., 2022). This is not unusual in wild species where stochastic environmental conditions will favor a bet‐hedging strategy that leads to a high genetic variability and a wide spectrum of natural phenotypes. Our three study species, *Viola pedatifida*, *V. sagittata*, and *V. lanceolata*, occur in prairie habitats and have both cleistogamous and chasmogamous flowers (self‐fertilizing closed and open flowers, respectively). Only *V. lanceolata* exhibits vegetative reproduction through stolons (De Vitis, personal observation). All three species are polyploid, which is common in Violets (Marcussen et al., 2012, 2022). For all three species, previous studies on seed dormancy and germination revealed that a cold stratification treatment increases germination rates compared to a control treatment (i.e., no stratification applied; De Vitis, unpublished data). However, the three species have been observed to have different germination behaviors: *V. lanceolata* usually shows a broader germination niche breadth (i.e., high germination proportions under a wider range of conditions) than the other two species, including considerable germination proportions even when freshly collected seeds are exposed to no cold stratification; *V. sagittata* shows a narrower niche breadth than *V. lanceolata*, showing intermediate germination result when exposed to the same cold stratification periods, and very low to no germination when freshly collected seeds are exposed to no cold stratification; *V. pedatifida* requires longer cold stratification than the other two species to achieve high levels of germination (De Vitis et al., in prep.; Kilgore et al., 2022).

### 2.2 Seed sourcing

In 2019, seeds of *V. lanceolata* and *V. sagittata* were provided by The Nature Conservancy Kankakee Sands Preserve Nursery (KS; Indiana, USA). At this nursery, beds for both species were established around 2015, using seeds collected from four local native populations. Seeds from these beds were harvested and used to grow a subsequent generation of plants in a greenhouse; then these potted plants were transferred outdoors. Seeds harvested from fruits of both chasmogamous and cleistogamous flowers were mixed and then were exposed to cold‐moist stratification for 60 days at 4°C. This seed mix was either used for next years' propagation material or for direct seeding in restorations. In addition, each year the nursery staff perform a small amount of wild collecting, depending on availability, to augment the levels of genetic diversity within the seed mix. Given the propagation practices carried out at this nursery, the number of maternal lines in the sourced seed sample was not known. The seed of *Viola pedatifida* was provided by the Lake County Forest Preserve Nursery (LCFP; Illinois, USA) and harvested in 2019 from plants grown in an in‐ground nursery production bed for one generation from seeds collected from a nearby wild population. All seeds were stored at +5°C until July 2019, when 100 randomly chosen seeds of each species were surface sterilized and sown into two Petri dishes per cold stratification treatment (25 seeds per replicate).

### 2.3 Cold‐moist stratification of subpopulations

We performed surface‐sterilization of all seeds by immersing and stirring the seeds into a 1% bleach solution for 2 min, followed by two consecutive 1‐min rinses in sterile water. Seeds were then sown in Petri dishes on 1.5% agar medium, sealed with parafilm, and exposed to cold stratification treatments at 0–3°C for different durations: for the short stratification, all species were stratified for 42 days, while for the long stratification seeds were stratified for 112 days for *V. sagitatta* and 84 days for *V. lanceolata* and *V. pedatifida*. These stratification lengths were determined to ensure a low but sufficient level of germination with short cold stratification, and to maximize germination with long stratification, based on conversations with practitioners.

At the end of the cold stratification periods, Petri dishes were transferred into an incubator maintained at 25/15°C (alternating day/night temperatures, 12/12 h cycle) and 12/12 photoperiod to trigger germination. From these two stratification duration treatments, we selectively generated two “cold stratification subpopulations” for each species. For the short subpopulation, we randomly selected 20 seeds that germinated within 2 weeks of moving to the incubator. A small proportion of seeds germinated while in cold stratification for *V. sagittata* and *V. pedatifida*, and none for *V. lanceolata*. For the long subpopulation, we discarded any individuals that germinated during the cold stratification, as we assumed they would pertain to the short or an intermediate subpopulation. By discarding seeds that germinated while in cold stratification, we simulated a real‐world application. Practitioners often stratify seeds in moist sand in the refrigerator during the winter to overcome dormancy prior to sowing seeds in flats in the spring (Nyberg & Haley, 2014); seeds that germinate in cold stratification are less likely to survive this process, as the radicle can easily be damaged when sowing. While the removal of some individuals that germinated during cold stratification may limit the full range genotypes and, therefore, genetic diversity observed in the long subpopulation, this experimental group should still sample a greater portion of the theoretical distribution of genotypes in the population compared to the short subpopulation and retain greater genetic diversity (Figure S1).

Following 2 weeks of warm stratification, more seeds had germinated in the long stratification treatment than the short for *V. pedatifida* (ca. 30% germination in short vs. 75% in the long) and *V. sagittata* (ca. 65%–80% germination in the short vs. 80%–90% in the long). Germination was similar between the short and long stratification treatments in *V. lanceolata* (85+%). The randomly selected germinated seeds were planted in individual plugs with peat media and transferred to a greenhouse with mist (day temperature of 21°C and night temperature of 18°C, with supplemental lighting from 6 AM to 10 PM to provide a long day photoperiod and mist running 3 s every 20 min from 4 AM to 11 PM). After about 1 week at these conditions, the seedlings were transplanted to larger pots and transferred to a second greenhouse without mist (day temperature of 19°C and night temperature of 17°C with supplemental lighting 6 AM to 10 PM). Here, the plants were watered every 1–2 days, and grown until seed production. From the 20 plants grown for each subpopulation, we randomly selected 13–14 individuals for the genetic analysis. The collection and experimental treatment of plants in this study followed relevant institutional, national and international guidelines and legislation.

### 2.4 Genomic sequencing, data processing, and statistical analyses

We genotyped individuals using double‐digestion restriction site‐associated sequencing (Peterson et al., 2012) and the STACKS v 2.2 pipeline (Catchen et al., 2011, 2013). First, we extracted genomic DNA from fresh leaf tissue using a DNeasy Plant Mini Kit (Qiagen, Venlo, Netherlands). Next, we used a modified double‐digestion restriction site‐associated sequencing, or ddRADseq, protocol (Peterson et al., 2012) to sample the genome of each species (Appendix S1). We used EcoRI and MspI restriction enzymes (New England Biolabs, Ipswich, MA, USA) to digest genomic DNA, after which we ligated adapters and used AMPureXP magnetic beads (Beckman Coulter, Indianapolis, IN, USA) to individually size‐select fragments between 500–900 bp (Appendix S1). We then amplified and cleaned up libraries and finally sequenced libraries using pair‐end 150‐bp sequencing on an Illumina NovaSeq 6000 (Appendix S1). We used STACKS v 2.2 (Catchen et al., 2011, 2013) to call single nucleotide polymorphisms (SNPs) de novo for each species (Appendix S1). We used a subset of individuals to optimize the STACKS parameters ‐m, ‐M, and ‐n (Paris et al., 2017). For different combinations of parameters, we compared patterns of genetic distance in Multidimensional Scaling Plots (MDS), the number of recovered variant sites, and measures of genetic diversity—optimal parameters are those that recover many SNPs, retain high levels of genetic diversity, and do not drastically change genetic relationships between individuals (Mastretta‐Yanes et al., 2015) (Appendix S1; Figures S2–S4; Tables S1–S3). We selected the default STACKS parameters (‐m 3, ‐M 2, and ‐n 2) for calling SNPs using the pipeline denovo_map.pl. For each species, all samples were included as being in the same population in the population map, required at least 35% of individuals in the population retain a locus for it to be processed (−r), a minimum minor allele frequency of 0.05 (‐‐min_maf), and one SNP was retained per stack to minimize linkage disequilibrium (‐‐write‐random‐snp).

We then generated three unique datasets for each species. First, we used VCFtools 0.1.14 (Danecek et al., 2011) to attain all loci that passed quality filtering. To quality filter SNPs, we exclude the following from the analysis: individuals with more than 80% missing data, loci with more than 45% missing data (‐‐max‐missing), and loci with an average minimum mean depth of coverage below 10× (‐‐min‐meanDP). Overall, SNPs had low to moderate read depth coverage and moderate to high percentage of missing data for individuals and sites for all species (Table S4). This group of high‐quality SNPs comprises the putatively “neutral SNPs” dataset for each species (Table S4), which we used to verify that individuals from the short and long subpopulations do not comprise distinct genetic clusters for each species. We evaluated population genetic structure using the first two axes of a scaled and centered principal components analysis (PCA) to evaluate genetic structure within each species using the package adegenet() (Jombart, 2008) in R v. 4.2.2 (R Core Team, 2022). In addition, we used the pairwise.neifst() function in the hierfstat package (Goudet, 2005) in R v. 4.2.2 (R Core Team, 2022) to measure Nei's F_ST_ between the short and long subpopulations of each species.

We then subset the neutral SNPs datasets to obtain two additional datasets. The second dataset that we generated for each species was the “annotated SNPs” dataset. We generated a FASTA file for the neutral SNPs dataset which we ran through the National Center for Biotechnology Information's (NCBI) database using the MegaBLAST® search program within the order of Violales (National Center for Biotechnology Information, 1988). The annotated SNPs are those that had a match, or hit, with an annotated sequence in the NCBI database and was associated with a specific protein (i.e., hits to uncharacterized loci, chloroplast or mitochondrial loci that were not related to a protein were excluded) (Table S5). If the top hit for a locus was not associated with a protein, the second best hit that was associated with a protein was selected. The third dataset for each species was the “shared, annotated SNPs” dataset. As we assumed physiological responses associated with short dormancy would be shared across taxa, we compared the NCBI database matches associated with proteins between species. For these shared annotated SNPs, we investigated the gene ontology for those proteins and determined the broad functional category of each protein based on the molecular function and biological processes listed in the PANTHER Classifications and UniProt databases as well as the literature (Table S6). The three datasets used in these analyses are subsets of one another meaning that each dataset represents a unique group of SNPs.

For each species we evaluated differences in genetic diversity between the short and long subpopulations using both the neutral SNPs datasets and the annotated SNPs datasets. Here, we are specifically interested in variation and diversity of alleles, rather than how alleles are arranged into genotypes. As such, for each locus, we measured gene diversity, or H_S_, which considers the frequency of alleles as well as their evenness in the population. We used the basic.stats() function in the hierfstat package (Goudet, 2005) in R v. 4.2.2 (R Core Team, 2022) to measure gene diversity for the short and long subpopulations of each species. To evaluate how the selection of short versus long moist cold stratification influences gene diversity, we tested for differences in H_S_ using a paired Wilcoxon rank sum test with Bonferroni correction using the function wilcox_test() in the package rstatix v.0.7.2 (Kassambara, 2023) (Table S7). For all comparisons the percent change in H_S_ was then calculated in loci in the long moist cold stratification subpopulation to the short subpopulation (e.g., [H_Slong_/H_Sshort_]/H_Slong_). Loci whose percent change was above 2 standard deviations above the mean percent change were then identified, suggesting that the change is greater than would be expected by chance and is, therefore, putatively a significant increase in percent change. If a locus was fixed for homozygosity (i.e., H_S_ = 0) in the short subpopulation but nonzero in the long subpopulation, then the percent change was considered as a significant increase from long to short (Table 2). If a locus was not present in at least two subpopulations, it was excluded from all analyses.

In addition, we measured rarified allelic richness (A_R_), or the rarefied count of alleles, for loci in each subpopulation using the allelic. richness() function in the hierfstat package (Goudet, 2005). For each species we rarefied A_R_ according to the subpopulation with the smallest sample size. We tested for differences in A_R_ using a paired Wilcoxon rank sum test with Bonferroni correction using the function wilcox_test() in the package rstatix v.0.7.2 (Kassambara, 2023). If a locus was not present in two or more subpopulations, it was excluded from all analyses.

## 3 RESULTS

**FIGURE 2:**
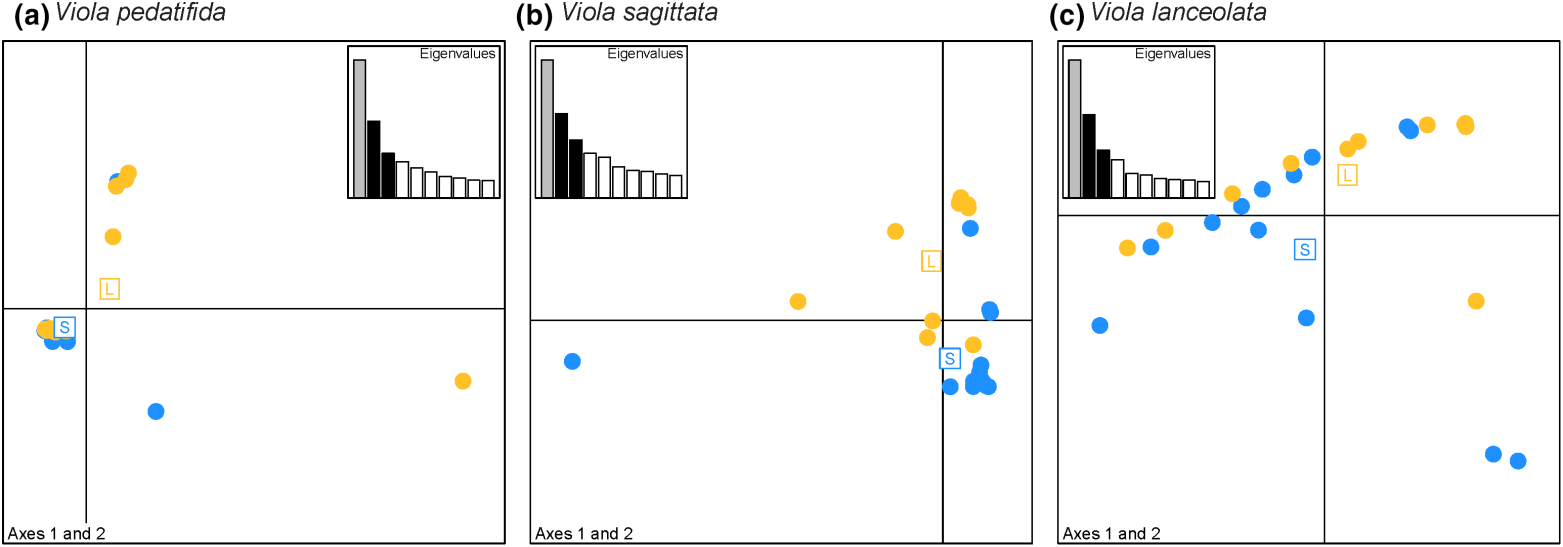
Population genetic structure of experimentally generated subpopulations of *Viola pedatifida*, *V. sagittata*, and *V. lanceolata*. Subpopulations were generated experimentally through either a short or long cold stratification pretreatment for alleviating physiological dormancy. The first two axes of the principal components analysis show each point as an individual and color corresponds to either the short (blue) or long (orange) subpopulation. Eigenvalues indicate the proportional amount of variance represented by each principal component.

Of the 75 individuals we included for genetic analyses, three *Viola lanceolata* and four *Viola sagittata* individuals failed to sequence and were excluded from analyses. As a result, sample sizes were reduced to 13 individuals in *Viola pedatifida*_Short_, 12 in *V. pedatifida*_Long_, 14 in *V. sagittata*_Short_, 9 in *V. sagittata*_Long,_ 13 in *V. lanceolata*_Short_, and 11 long *V. lanceolata*_Long_. The putatively neutral single nucleotide polymorphisms (SNPs) datasets included 2821 quality filtered SNPs for *V. pedatifida*, 2042 for *V. sagitatta*, and 1662 for *V. lanceolata*. For the neutral SNPs datasets for each species, individuals exhibited low to moderate amounts of missing data (average range = 9.93%–35.93%) and low to moderate read depth coverage (average range = 20.61–21.15 X) (Table S4). In total we identified 382 SNPs as occurring in an annotated region for *V. pedatifida*, 332 for *V. sagitatta*, and 197 for *V. lanceolata*. For the annotated SNPs datasets for each species, individuals exhibited low to moderate amounts of missing data (average range = 8.89%–31.94%) and low to moderate read depth coverage (average range = 23.74–25.19 X) (Table S4). For all species, individuals from the short and long subpopulations clustered together on both axes of the principal components analysis (Figure 2a–c). Nei's pairwise F_ST_ between short and long subpopulations were 0.070 for *V. pedatifida*, 0.083 for *V. sagitatta*, and 0.068 for *V. lanceolata*.

**FIGURE 3:**
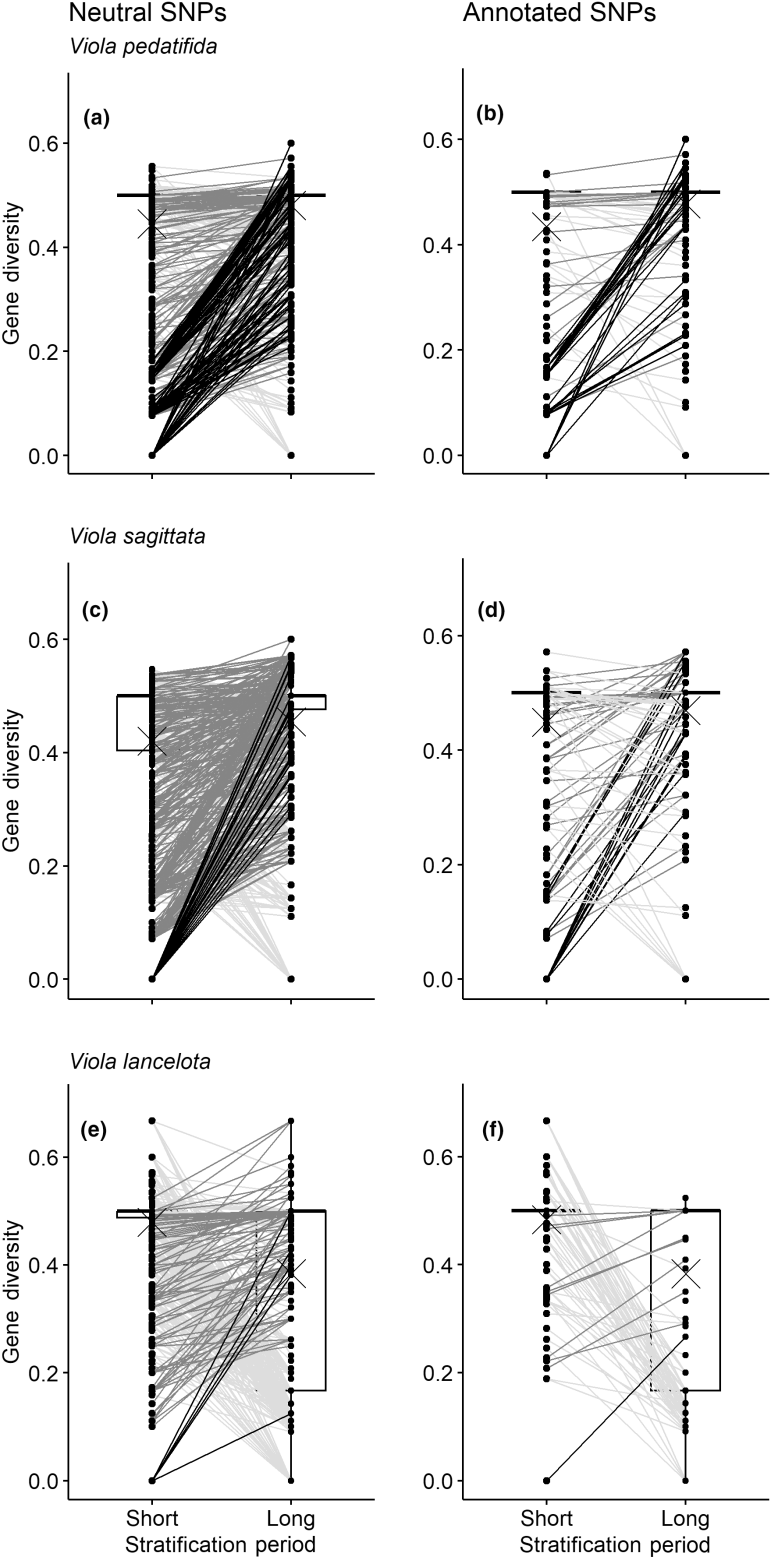
Paired gene diversity of loci in two subpopulations propagated through either a short or a long moist cold‐stratification for three species of violets. Box plots show gene diversity (H_S_) of all, putatively neutral single nucleotide polymorphisms (a, c, e) and those that are annotated (b, d, f), with the mean denoted by the cross. Line color indicates if a locus experienced a significant increase (black), increase (dark gray) or decrease (light gray) in the percent change in gene diversity between the long and short subpopulations. Loci with no percent change from long to short subpopulations are not shown.

When using the neutral SNPs dataset to compare differences in gene diversity (H_S_), we found that in *V. pedatifida* and *V. sagitatta*, gene diversity was significantly higher in the subpopulations exposed to the long cold‐stratification compared to those exposed to the short stratification period, but the effect of stratification period was small (Table 1, Figure 3a,c; Table S7). For *V. pedatifida* and *V. sagitatta*, 17.6% and 26.4% of neutral SNPs had higher genetic diversity in the subpopulations exposed to the long stratification compared to the short stratification subpopulation (Figure 3a,c and Table 2). In contrast, *V. lanceolata* showed significantly higher gene diversity in short‐stratified than the long‐stratified subpopulation for neutral SNPs with a strong effect of stratification period (Table 1; Figure 3e and Table S7). Regardless, 8.5% of the loci were more variable in the long compared to short subpopulation for *V. lanceolata* (Figure 3e and Table 1). For *V. pedatifida*, *V. sagitatta*, and *V. lanceolata*, 7.9%, 16.4%, and 27.7% of neutral SNPs, respectively, had lower genetic diversity in the subpopulations exposed to the long stratification compared to the short stratification subpopulation (Table 2). For all species, the majority of neutral SNPs did not experience a change in gene diversity (Table 2). In addition, we found that rarefied allelic richness (A_R_) was larger in the long subpopulation for neutral SNPs in *V. pedatifida* and *V. sagitatta* with a moderate and large effect size of stratification time, respectively (Table 1 and Table S7). Rarefied allelic richness (A_R_) was significantly greater in the short subpopulation for *V. lanceolata*, but the effect size was small (Table 1 and Table S7).

**TABLE 1:** Results of statistical tests evaluating differences between gene diversity (H_S_) and rarefied allelic richness (A_R_) in subpopulations of three species of Violets germinated under a short or long period in moist cold stratification.

Species	SNP category	*N*	Gene diversity (H_S_)				Rarefied allelic richness (A_R_)			
			Sub population	*p*‐Value	Test statistic (V)	Effect size	Sub population	*p*‐Value	Test statistic (V)	Effect size
*Viola pedatifida*	Neutral	2821	Long	<0.0001	211,224	0.198	Long	<0.0001	114,908	0.344
	Annotated	382	Long	<0.0001	3124	0.139	Long	<0.0001	2343	0.359
*Viola sagitatta*	Neutral	1904	Long	<0.0001	227,600	0.175	Long	<0.0001	361,952	0.577
	Annotated	312	Long	0.0079	2996	0.121	Long	<0.0001	6708	0.484
*Viola lanceolata*	Neutral	1463	Short	<0.0001	24,452	0.396	Short	0.028	717	0.0142
	Annotated	197	Short	<0.0001	129	0.45	Short	0.0258	8.5	0.151

*Note*: Shown are species names, the category of single nucleotide polymorphism (SNP) as either one with a putative biological function (i.e., annotated SNP) or not (i.e., neutral SNP) and the number of SNPs in each category (N). Also shown is the subpopulation with the greater value for each measure as well as the *p*‐value, test statistic, and effect size of statistical tests.

**TABLE 2:** Summary of the percent change in gene diversity (H_S_) in subpopulations of three species of violets propagated under long moist cold stratification to those propagated under short cold stratification.

Species	SNP category	*N*	Fixed homozygosity	Average percent change ±1 SD	Increase	Significant increase	No change	Decrease	Significant decrease
*Viola pedatifida*	Neutral	2821	10	0.075 ± 0.27	176 (6.2%)	311 (11.0%)	2101 (74.5%)	194 (6.9%)	29 (1.0%)
	Annotated	442	3	0.056 ± 0.26	21 (4.8%)	44 (10.0%)	336 (76.0%)	34 (7.7%)	7 (1.5%)
*Viola sagittata*	Neutral	1904	51	0.045 ± 0.40	435 (22.9%)	67 (3.5%)	1090 (57.35)	227 (11.9%)	85 (4.5%)
	Annotated	312	8	0.026 ± 0.33	43 (13.8%)	14 (4.5%)	217 (69.6%)	27 (8.7%)	11 (3.5%)
*Viola lanceolata*	Neutral	1662	7	−0.64 ± 1.3	150 (9.0%)	7 (0.42%)	753 (45.3%)	128 (7.7%)	332 (20.0%)
	Annotated	197	1	−0.72 ± 1.38	10 (5.1%)	1 (0.51%)	125 (63.5%)	46 (23.4%)	16 (8.1%)

*Note*: Shown are the species name, the category of single nucleotide polymorphism (SNP) as either one with a putative biological function (i.e., annotated SNP) or not (i.e., neutral SNP), and the number of loci in each SNP category. In addition, percent change in gene diversity from the short to long subpopulations is described by the average percent change in (H_S_), the number of SNPs fixed for homozygosity in the short subpopulation, as well as the number of loci with a likely significant increase, an increase, no change, a decrease, and a likely significant decrease in percent change from the long to short subpopulations. The proportion of total SNPs is shown in parentheses.

Similarly for the annotated loci, gene diversity (H_S_) was significantly higher in the long‐stratified subpopulation in *V. pedatifida* and *V. sagitatta* compared to the subpopulation experiencing short cold stratification with a small effect of stratification period (Table 1, Figure 3b,d and Table S7). For these species, 14.7% and 18.3% of annotated SNPs had higher gene diversity in the long‐stratified subpopulation (Figure 3b,d and Table 2). Again, gene diversity in the short‐stratified *V. lanceolata* subpopulation was significantly higher than the long‐stratified subpopulation with stratification time having a moderate effect (Table 1 and Table S7), but 5.6% of annotated SNPs were variable in the long subpopulation (Figure 3f and Table 2). For *V. pedatifida*, *V. sagitatta*, and *V. lanceolata*, 9.2%, 12.5%, and 31.5% of annotated SNPs, respectively, had lower genetic diversity in the subpopulations exposed to the long stratification compared to the short stratification subpopulation (Table 2). For all species, the majority of annotated SNPs did not experience a change in gene diversity (Table 2). Rarefied allelic richness (A_R_) was larger in the long subpopulation for annotated SNPs of *V. pedatifida* and *V. sagitatta* and the effect of stratification time was moderate for *V. pedatifida* (Table 1). However, A_R_ in the short‐stratified *V. lanceolata* subpopulation was significantly higher than the long‐stratified subpopulation with stratification time having a small effect (Table 1 and Table S7).

Of the annotated SNPs, 537 were matched to the same protein in at least two species and were related to a variety of molecular functions and biological processes (Table 3 and Table S5). Of these SNPs, 220 were found in *V. pedatifida*, 189 in *V. sagitatta*, and 112 in *V. lanceolata*. In total, gene diversity was lower in 15.0% of shared annotated loci in *V. pedatifida*, 15.3% in *V. sagitatta*, and 4.5% of loci in *V. lanceolata* in the short subpopulation. These shared annotated SNPs were not restricted to genes associated with dormancy or seed germination but ranged in molecular functions and biological processes from plant development and growth to defense (Table 3 and Table S6).

**TABLE 3:** Summary of the general protein function and the number of shared annotated single nucleotide polymorphisms (SNPs) in each annotated category.

Species	Broad annotated categories of proteins	Number of shared annotated SNPs
*Viola pedatifida*	Cellular function	6
	Photosynthesis	4
	Plant defense	1
	Plant growth/development	11
	Protein synthesis	2
	Respiration	2
	Response to environment	1
	Unknown	1
*Viola sagittata*	Cellular function	3
	DNA replication	1
	Photosynthesis	2
	Plant defense	1
	Plant growth/development	8
	Protein synthesis	8
	Respiration	4
	Unknown	1
*Viola lanceolata*	Cellular function	2
	Photosynthesis	1
	Protein synthesis	1
	Respiration	1

*Note*: Shown are SNPs with decreased genetic in the subpopulation exposed to a short‐moist cold stratification. Shared annotated SNPs were those in the same gene pathway in at least two species.

## 4 DISCUSSION

In this study we demonstrate that production practices used to generate plant materials of wild species used in restoration can decrease genetic diversity in both putatively neutral and annotated loci. Specifically, for two out of three species, we found that using a short stratification fixes homozygosity in many loci compared to a longer stratification, which maintained higher genetic diversity. However, the impact was not equivalent across all three study species. Despite belonging to the same genus (*Viola*), the germination requirements of each taxon influenced the effect of production practices on genetic diversity. We found that the two species with a deeper physiological dormancy, *V. pedatifida* and *V. sagitatta*, are more likely to be impacted by short cold stratification conditions, as they had higher genetic diversity in the long subpopulation. On the other hand, *V. lanceolata* is a species with less stringent dormancy requirements and exhibited higher genetic diversity in the short subpopulation. For *V. pedatifida* and *V. sagitatta*, our data showed that some loss of genetic diversity was in loci which have known biological function and may represent a loss of evolutionary potential for these gene regions. We verified that differences in genetic diversity were not attributed to population genetic structuring, as there was no structuring between the short and long groups and measures of genetic differentiation were relatively low. In addition, we found that stratifying seeds prior to sowing, as in Nyberg and Haley (2014), is more likely to truncate genetic diversity as seeds that germinate in cold stratification are less likely to survive because their radicles can be damaged in the transfer from the stratification medium to the growing pot. An alternative practice to germinate and propagate plants, beside the one we described an applied in this study, is to sow seeds in plug trays prior to placing in cold stratification (see Sollenberger et al., 2022). This approach is less common because it requires a much larger refrigerated space; however, seeds that germinate in cold stratification may be equally as likely to survive as those that germinate after being removed from cold stratification because they germinate directly in the plug where they will grow.

The germination ecology of each species was related to the effects that short versus long stratification had on genetic diversity, underscoring the importance of considering the species biology when predicting the effects of production practices. Both *V. sagittata* and *V. pedatifida* showed lower genetic diversity in the short subpopulation. For both taxa, seeds germinated at higher rates when exposed to a long cold stratification pretreatment than a shorter cold stratification (De Vitis et al. in prep.; Kilgore et al., 2022). Given this almost linear correlation between the duration of the cold stratification and the proportion of germinating seeds, selecting for a short cold stratification will include fewer individuals and is, therefore, likely to impose both a genetic bottleneck and selection for genotypes with shallower physiological dormancy leading to a decrease in genetic diversity (e.g., Figure 1c). Conversely, using a long cold stratification would likely translate to a cumulative effect whereby individuals that can germinate under short and long cold stratification are included in the plant materials produced for reintroduction resulting in higher genetic diversity (e.g., Figure 1b). In contrast, *V. lanceolata* had higher genetic diversity in the short subpopulation and it is the only species whose wild sourced seeds can germinate at high rates (40%) with no cold stratification pretreatment (De Vitis et al. in prep.; Kilgore et al., 2022), which may help explain why there was little effect of the duration of cold stratification on genetic diversity. Given this larger germination niche breadth, we might expect that genetic diversity would be equivalent in the short and long subpopulations, as the same genotypes should be represented in both groups. However, as we observed lower genetic diversity in the long subpopulation of *V. lanceolata*, we suspect that either we unknowingly sampled a group of individuals with lower genetic diversity (i.e., sampling error) in the long subpopulation or that there is a biological process underpinning the lower genetic diversity in this subpopulation for this taxon. Importantly, we recognize that this work is limited by a small sample size as well as a lack of direct tests of which mechanisms (i.e., selection and drift) are resulting in evolutionary change. In addition, we highlight that our measure of gene diversity (H_S_) may overestimate genetic diversity as our study species are polyploids and as a result exhibit high rates of heterozygosity due to the presence of paralogs. We recommend that future research explicitly test the relative effect of artificial selection and genetic bottlenecks on genetic diversity in the production process using a broader sampling scheme and more thorough replication.

The decreased genetic diversity in putatively neutral and functional loci highlights that it is possible to significantly lower gene diversity within a single generation through the application of rapid propagation practices. Similarly, in analyzing the genotypic and phenotypic changes in five wild plant species during multiple generations under cultivation, Nagel et al. (2019) found that in two species, a significant proportion of alleles were lost during cultivation despite not detecting decreased overall genetic variability. While the unintentional loss of genetic diversity through propagation has been a longstanding concern of restoration ecologists (Basey et al., 2015; Espeland et al., 2017; Havens et al., 2004), most studies have documented genetic shifts rather than decreases in genetic diversity (Dyer et al., 2016; Nagel et al., 2019; St. Clair et al., 2020). On the other hand, large scale seed production for restoration has not led to major changes in genetic diversity in other studied species (Kucera et al., 2022; St. Clair et al., 2020), even in those that experience dormancy (Conrady et al., 2022). In some cases, propagation practices can lead to ex situ populations harboring greater levels of genetic diversity compared to in situ populations (Conrady et al., 2022; St. Clair et al., 2020), demonstrating that cultivation, when applied appropriately, can benefit species that are to be restored in harsh in situ conditions (Chivers et al., 2016). This suggests that when done properly, the production of plant materials for restoration can safeguard genetic diversity and improve species' ability to successfully be reintroduced to native environments, emphasizing the need for the use of best practices that consider nuanced, species‐specific biological requirements such as pretreatments for alleviating physiological dormancy (Basey et al., 2015; Espeland et al., 2017). Together with past studies, our work further stresses the importance of considering how production practices may unintentionally impact genetic diversity of focal species.

The lower genetic diversity in shared, annotated loci demonstrates that the application of a short cold stratification can limit the evolutionary potential of the material used in reintroduction for genes related to a range of functions. As expected, many of the annotated loci identified in more than two species are related to plant development pathways that are impacted by selection for rapid germination. For example, some of these loci are likely associated with NADH dehydrogenase subunit 1 and subunit 7, both of whose transcripts interact with pentatricopeptide repeat proteins to influence ABA hypersensitivity, seed dormancy, and delayed germination (Hammani et al., 2011; Lee et al., 2017; Nonogaki, 2019). Another shared, annotated locus with decreased gene diversity in *V. sagittata* was likely related to the DOT2 gene region which encodes an SART‐1 family protein that is related to seedling development (Petricka et al., 2008) and mutations in this gene can result in dwarf plants with reduced root length (Casson et al., 2005). However, several of annotated loci with lower gene diversity in the short subpopulation were not related to germination and development and showed more generalized cellular functions, suggesting that selective sweeps could have cascading consequences in decreasing the adaptive potential in a variety of gene pathways unrelated to dormancy and development (Barghi & Schlötterer, 2020; Muralidhar & Veller, 2022). Such a pattern could also be explained by a genetic bottleneck, emphasizing the need for additional research in this area. Retaining high levels of genetic diversity across multiple functional regions of the genome is likely to be an important advantage for natural populations that are exposed to a broad range of abiotic conditions (Gorecki et al., 2012; Long et al., 2015). This study emphasizes the need for more research that investigates the consequences of decreased variation in specific gene regions to understand the implications that production practices can have on plant reintroduction efforts.

Taken together, this work underscores the necessity to consider that micro‐evolutionary processes continue under production practices and when generating plant material that is used in restoration, these practices may impact genetic diversity of the end product. Given the variable environmental conditions that ecosystems will face, highlighted by the ongoing effects of climate change, retaining genetic diversity in reintroduced populations is vital to their resilience and adaptative potential (Harris et al., 2006; Willi et al., 2022). Due to the uncertainty in predicting future climates, we are likely to maximize the success of restoration efforts when using genetic material that enables populations to adapt to multiple, equally probable climate change scenarios (Crowe & Parker, 2008). If not, there is a high risk of wasting biological and economic resources, and of compromising confidence in conservation actions if cultivation practices result in reintroduced populations that are unable to persist in the wild. Furthermore, the conservation value of these species may continue to decline if artificial selection is imposed over multiple generations, especially given that selecting for fast germination led to fixed homozygosity in a subset of loci in all species in one generation. We recommend integrating research on species‐specific requirements for seed dormancy breakage and germination into commercial plant propagation practices and applying the appropriate stratification length to each species.

## CONFLICT OF INTEREST STATEMENT

There are no conflicts of interest to declare.

## Supporting information


Data S1.

## Data Availability

Relevant data and code are publicly available on Dryad (https://datadryad.org/stash/share/0e_mT‐Xix‐vAVvQBb3gtRRvuaLMT4Uh875yEbXQMfA0).
