# Supplementary material for: Species‐specific effects of production practices on genetic diversity in plant reintroduction programs
Source: Evol Appl. 2023 Nov 20;16(12):1956–68. doi: 10.1111/eva.13614 (PMC10739063; doi:10.1111/eva.13614)
Supplement: Supplementary file 1 — Data S1. [file EVA-16-1956-s001.zip › Supplementary_Information.docx]

**Appendices**

Appendix A. Detailed methods for genomic sequencing and data processing. We digested genomic DNA of 96 samples using the EcoRI and MspI restriction enzymes (New England Biolabs, Ipswich, MA, USA). We created two libraries using 48 adapters with unique barcodes specific to the EcoRI cutsite and a common MspI adapter (i.e., each group of 48 samples was a distinct library). We used AMPureXP magnetic beads (Beckman Coulter, Indianapolis, IN, USA) to individually size-select fragments between 500-900 bp. Then, we quantified the concentration of each sample using a Qubit fluorometer (Waltham, MA, USA). We pooled samples within each library based on sample concentration so that each library had four pools. Next, we amplified each pool using a multiplexing MspI primer. We used two distinct multiplexing primers that were specific to each library. We combined pools within libraries using equal DNA mass from each to create the final sequencing libraries. Final sequencing libraries were checked for the appropriate size distribution on an Agilent BioAnalyzer (Santa Clara, CA, USA) with the High Sensitivity DNA assay and concentration were quantified using a Qubit fluorometer (Waltham, MA, USA). We then performed pair-read 150-bp sequencing on an Illumina NovaSeq 6000 platform at the Northwestern School of Medicine Sequencing Core (Chicago, IL, USA).

We de-multiplexed and filtered sequencing reads using STACKS v 2.2 with process_radtags and removed barcodes with up to two mismatches (-adapter_mm), rescued barcodes with up to one mismatch (-r), cleaned data to remove any uncalled bases (-c), discarded reads with low quality scores by default settings (-q), and trimmed all reads to 100 bases (-t). The parameters -m, -M, and -n were optimized in the denovo_map.pl pipeline using four individuals from the short and long experimental groups. For all species, the different combinations of the STACKS parameters resulted in comparable patterns of genetic distance in the MDS plots, similar number of SNPs, and near equivalent levels of genetic diversity (Mastretta‐Yanes et al. 2015) (Supplementary Figures 1-3 and Supplementary Tables 1-3). Therefore, the default parameters for each species were used, which are generally appropriate within a species (Paris, Stevens, & Catchen 2017). For the final SNP calling, the denovo_map.pl pipeline uses all samples to build the catalog. Short and long subpopulations may have been sourced from a combination of the same and different original populations.


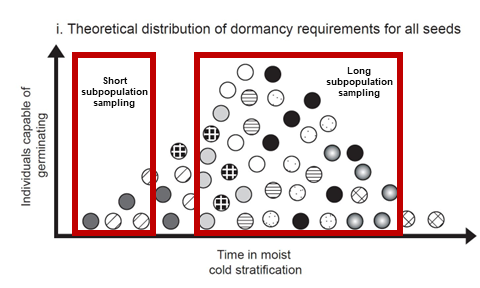


**Supplementary Figure S1.** Presumed experimental sampling approach for short and long subpopulations used in genetic analysis. If genotype is associated with the capacity to germinate based on time in cold stratification, the short subpopulation is assumed to have sampled a relatively small portion of all genotypes in the population. If genotype is associated with the capacity to germinate based on time in cold stratification, the long subpopulation is assumed to have sampled a relatively greater portion of all genotypes in the population. For *V. pedatifida* and *V. sagittata* a few seeds germinated while in cold stratification and were excluded from the long subpopulation; hence there are some genotypes that might have been excluded from this group.


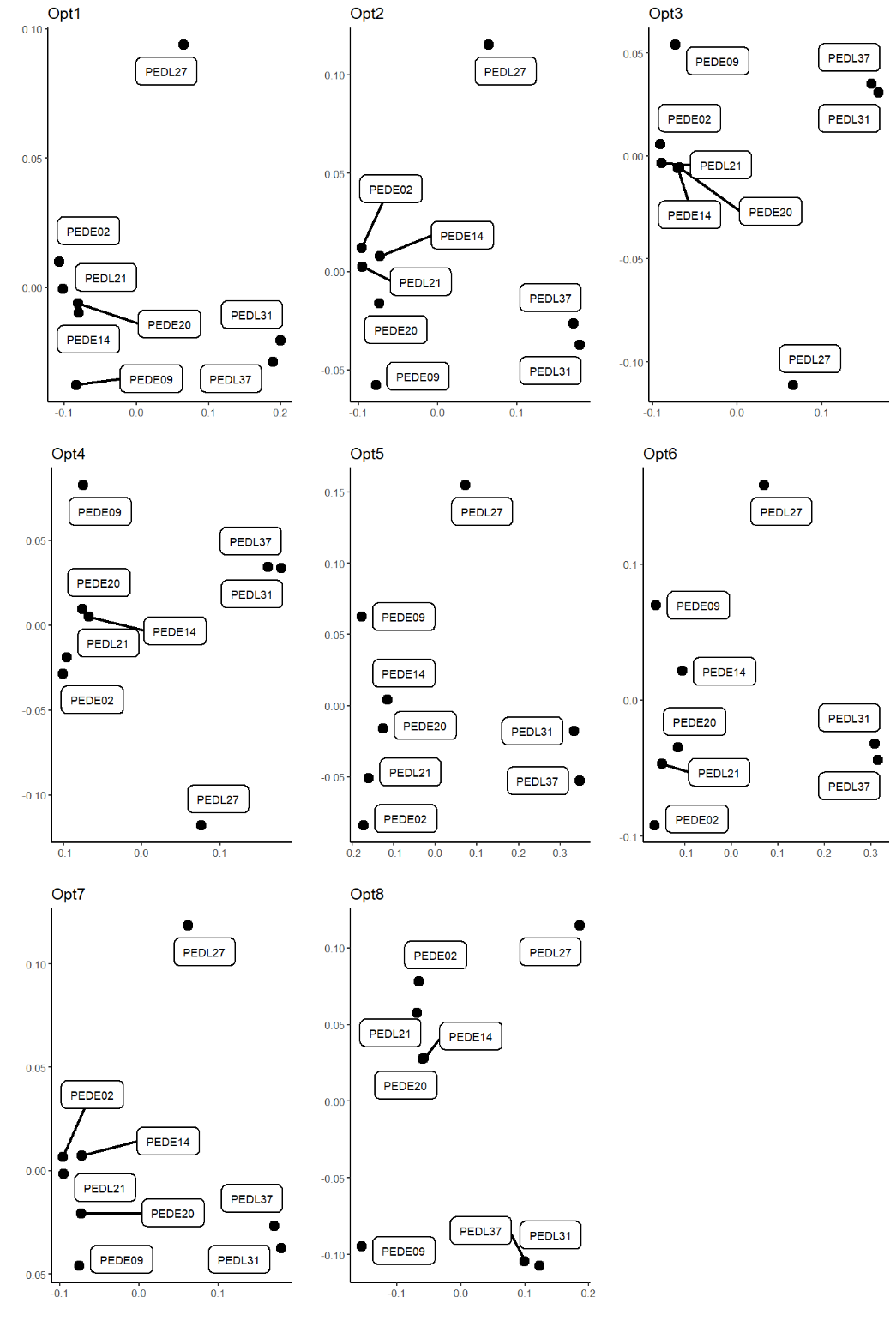


Supplementary Figure 2. Optimization of three core STACKS parameters, -m, -M, and -n, and the associated metric multidimensional scaling plots (MDS) for each combination of parameters for *Viola pedatifida*. Parameters were optimized using four individuals from the short and long moist-cold stratification conditions. The x- and y-axes represent the first and second components of the MDS. The parameter combination for each optimization can be found in Table S1.


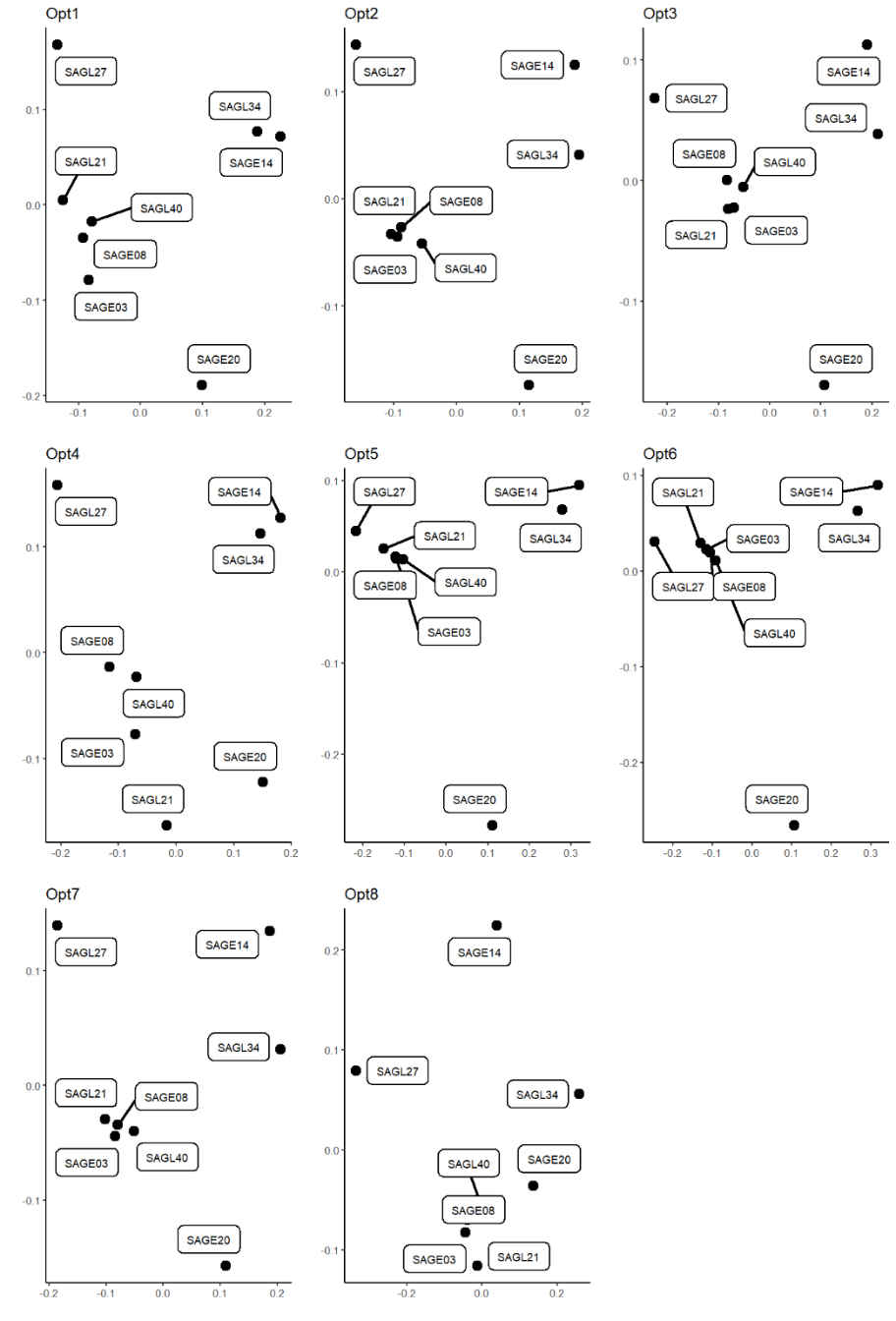


**Supplementary Figure 3**. Optimization of three core STACKS parameters, -m, -M, and -n, and the associated metric multidimensional scaling plots (MDS) for each combination of parameters for *Viola sagittata*. Parameters were optimized using four individuals from the short and long moist-cold stratification conditions. The x- and y-axes represent the first and second components of the MDS. The parameter combination for each optimization can be found in Table S2.


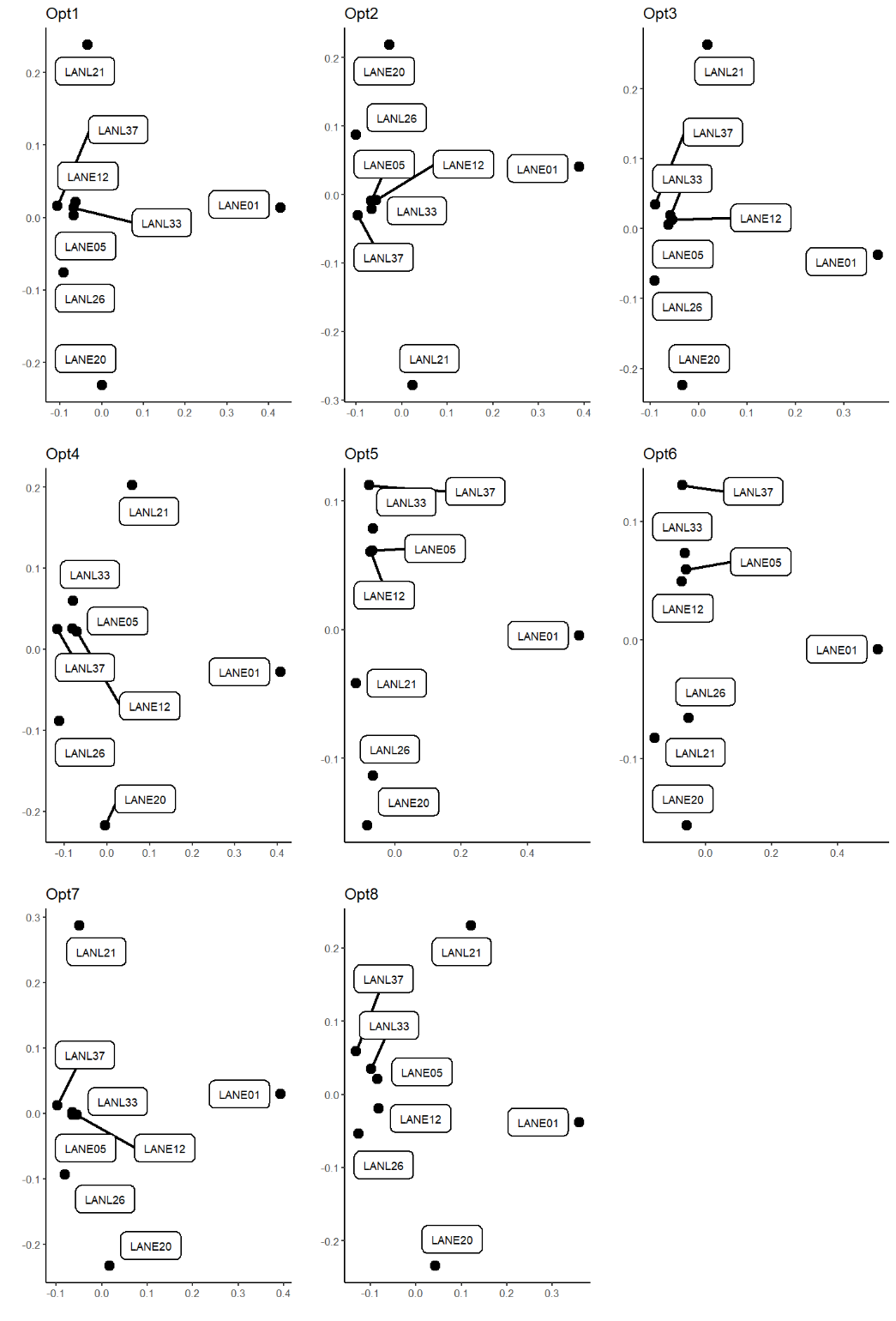


Supplementary Figure 4. Optimization of three core STACKS parameters, -m, -M, and -n, and the associated metric multidimensional scaling plots (MDS) for each combination of parameters for *Viola lanceolata*. Parameters were optimized using four individuals from the short and long moist-cold stratification conditions. The x- and y-axes represent the first and second components of the MDS. The parameter combination for each optimization can be found in Table S3.

Supplementary Table 1. Optimization of three core STACKS parameters and the associated measures of genetic diversity for each combination of parameters for *Viola pedatifida*. Parameters were optimized using four individuals from the short and long moist-cold stratification conditions.

|  | Parameters tested | | | Variant positions (avg over populations) | | | | All positions (avg over pops) |
| --- | --- | --- | --- | --- | --- | --- | --- | --- |
| Name | -m | -M | -n | He | SE | pi | SE | Polymorph. Sites |
| Opt1 | 3 | 2 | 2 | 0.39 | 0.0017 | 0.56 | 0.0028 | 9981 |
| Opt2 | 4 | 2 | 2 | 0.40 | 0.0016 | 0.58 | 0.0028 | 10475 |
| Opt3 | 5 | 2 | 2 | 0.40 | 0.0016 | 0.59 | 0.0027 | 10868 |
| Opt4 | 3 | 3 | 3 | 0.39 | 0.0018 | 0.57 | 0.0032 | 8446 |
| Opt5 | 3 | 1 | 1 | 0.36 | 0.0021 | 0.54 | 0.0036 | 6705 |
| Opt6 | 4 | 1 | 1 | 0.37 | 0.0021 | 0.56 | 0.0035 | 7011 |
| Opt7 | 4 | 2 | 2 | 0.40 | 0.0016 | 0.58 | 0.0028 | 10479 |
| Opt8 | 3 | 4 | 4 | 0.39 | 0.0020 | 0.59 | 0.0035 | 7186 |

Supplementary Table 2. Optimization of three core STACKS parameters and the associated measures of genetic diversity for each combination of parameters for *Viola sagittata*. Parameters were optimized using four individuals from the short and long moist-cold stratification conditions.

|  | Parameters tested | | | Variant positions (avg over populations) | | | | All positions (avg over pops) |
| --- | --- | --- | --- | --- | --- | --- | --- | --- |
| Name | -m | -M | -n | He | SE | pi | SE | Polymorph. Sites |
| Opt1 | 3 | 2 | 2 | 0.36 | 0.0015 | 0.49 | 0.0024 | 13154 |
| Opt2 | 4 | 2 | 2 | 0.36 | 0.0015 | 0.50 | 0.0024 | 13455 |
| Opt3 | 5 | 2 | 2 | 0.37 | 0.0015 | 0.51 | 0.0024 | 13590 |
| Opt4 | 3 | 3 | 3 | 0.35 | 0.0017 | 0.49 | 0.0027 | 10940 |
| Opt5 | 3 | 1 | 1 | 0.35 | 0.0018 | 0.49 | 0.0028 | 10035 |
| Opt6 | 4 | 1 | 1 | 0.35 | 0.0017 | 0.50 | 0.0028 | 10199 |
| Opt7 | 4 | 2 | 2 | 0.36 | 0.0015 | 0.50 | 0.0024 | 13463 |
| Opt8 | 3 | 4 | 4 | 0.35 | 0.0019 | 0.50 | 0.0030 | 9073 |

Supplementary Table 3. Optimization of three core STACKS parameters and the associated measures of genetic diversity for each combination of parameters for *Viola lanceolata*. Parameters were optimized using four individuals from the short and long moist-cold stratification conditions.

|  | Parameters tested | | | Variant positions (avg over populations) | | | | All positions (avg over pops) |
| --- | --- | --- | --- | --- | --- | --- | --- | --- |
| Name | -m | -M | -n | He | SE | pi | SE | Polymorph. Sites |
| Opt1 | 3 | 2 | 2 | 0.24 | 0.0020 | 0.34 | 0.0031 | 6930 |
| Opt2 | 4 | 2 | 2 | 0.25 | 0.0021 | 0.36 | 0.0032 | 6994 |
| Opt3 | 5 | 2 | 2 | 0.26 | 0.0021 | 0.38 | 0.0033 | 7095 |
| Opt4 | 3 | 3 | 3 | 0.24 | 0.0023 | 0.34 | 0.0035 | 5538 |
| Opt5 | 3 | 1 | 1 | 0.22 | 0.0023 | 0.33 | 0.0036 | 5096 |
| Opt6 | 4 | 1 | 1 | 0.24 | 0.0023 | 0.36 | 0.0037 | 5299 |
| Opt7 | 4 | 2 | 2 | 0.25 | 0.0021 | 0.36 | 0.0032 | 6991 |
| Opt8 | 3 | 4 | 4 | 0.24 | 0.0025 | 0.35 | 0.0040 | 4538 |

Supplementary Table 4. Quality information for filtered single nucleotide polymorphisms (SNPs) for three species of violets: *Viola pedatifida*, *V. sagittata*, and *V. lanceolata*.

|  |  |  |  | **Individual** | | | | **Site** | | | |
| --- | --- | --- | --- | --- | --- | --- | --- | --- | --- | --- | --- |
| **Species** | **N** | **SNP Type** | **# SNPs** | **Avg. Missing Data (%)** | **Missing Range (%)** | **Avg. Site Depth (X)** | **Depth Range (X)** | **Avg. Missing Data (%)** | **Missing Range (%)** | **Avg. Site Depth (X)** | **Depth Range (X)** |
| *Viola pedatifida* | 25 | All | 2821 | 9.93 ± 8.09 | 3.15 – 41.19 | 21. 36 ± 7.55 | 11.84 - 38.95 | 9.93 ± 9.79 | 0.00 - 52.00 | 21.53 ± 12.81 | 10.24 - 230.52 |
|  | 25 | Annotated | 382 | 8.89 ± 8.85 | 1.83 - 45.29 | 25.19 ± 8.73 | 13.65 - 46.36 | 8.89 ± 9.34 | 0.00 - 52.00 | 25.56 ± 21.56 | 11.13 - 230.11 |
| *Viola sagittata* | 23 | All | 2042 | 12.26 ± 13.60 | 2.11 - 58.08 | 20.61 ± 8.75 | 9.04 - 40.19 | 12.26 ± 10.96 | 0.00 - 52.00 | 21.33 ± 15.20 | 10.61 - 311.00 |
|  | 23 | Annotated | 332 | 10.83 ± 13.57 | 6.02 - 59.64 | 23.74 ± 10.31 | 8.74 - 47.86 | 10.83 ± 10.60 | 0.00 - 47.83 | 24.44 ± 18.39 | 10.45 - 207.26 |
| *Viola lanceolata* | 24 | All | 1662 | 35.93 ± 23.28 | 6.08 - 75.53 | 21.15 ± 10.64 | 10.59 - 49.16 | 35.94 ± 16.42 | 0.00 - 58.33 | 21.65 ± 14.50 | 16.34 - 338.5 |
|  | 24 | Annotated | 197 | 31.94 ± 23.37 | 4.57 - 82.23 | 24.41 ± 12.99 | 11.83 - 61.47 | 31.94 ± 16.76 | 0.00 - 58.33 | 25.04 ± 17.24 | 10.15 - 196.6 |

Supplementary Table 5 (separate file). Excel spreadsheet of single nucleotide polymorphisms with matches, or hits, in the National Center for Biotechnology Information’s database using the BLAST® megablast search function to search within the order of Violales.

Supplementary Table 6 (separate file). Excel spreadsheet of the gene ontology of functional SNPs shared among at least two species.

Supplementary Table 7. Average gene diversity for three species of violets germinated under different cold stratification conditions. Shown is average gene diversity (H_S_ ± 1SD) and rarefied allelic richness (A_R_) for all single nucleotide polymorphisms (All) and those with a potential biological function (Annotated). Seeds were germinated with either a short or long cold stratification.

| **Species** | **SNP type** | **Condition** | **Average Hs** | **Average A_R_** |
| --- | --- | --- | --- | --- |
| *Viola pedatifida* | All | Short | 0.45 ± 0.13 | 1.38 ± 0.53 |
|  |  | Long | 0.48 ± 0.07 | 1.45 ± 0.59 |
|  | Annotated | Short | 0.45 ± 0.48 | 1.34 ± 0.50 |
|  |  | Long | 0.48 ± 0.08 | 1.40 ± 0.56 |
| *Viola sagittata* | All | Short | 0.42 ± 0.45 | 1.43 ± 0.51 |
|  |  | Long | 0.45 ± 0.11 | 1.63 ± 0.67 |
|  | Annotated | Short | 0.45 ± 0.12 | 1.40 ± 0.51 |
|  |  | Long | 0.47 ± 0.10 | 1.50 ± 0.61 |
| *Viola lanceolata* | All | Short | 0.48 ± 0.09 | 1.86 ± 0.83 |
|  |  | Long | 0.38 ± 0.17 | 1.86 ± 0.83 |
|  | Annotated | Short | 0.48 ± 0.09 | 1.75 ± 0.85 |
|  |  | Long | 0.38 ± 0.17 | 1.71 ± 0.83 |
